# Agronomic Response of Silage Maize to Varying Irrigation Water and Nitrogen Levels Under Subsurface Drip Irrigation: Implications for Yield and Water Use Productivity

**DOI:** 10.3390/plants15172734

**Published:** 2026-09-07

**Authors:** Filiz Akin, Köksal Aydinşakir, Ömer Özbek, Şekip Erdal, Gökhan Uçar, Mehmet Pamukçu, Mehmet Kocatürk, Cihan Karaca, Şerife Gülden Yilmaz, Dursun Büyüktaş, Bilal Cemek

**Affiliations:** 1 Department of Soil and Water Resources, Batı Akdeniz Agricultural Research Institute, 07100 Antalya, Türkiye; koksal.aydinsakir@tarimorman.gov.tr (K.A.); omer.ozbek@tarimorman.gov.tr (Ö.Ö.); gokhan.ucar@tarimorman.gov.tr (G.U.); 2 Department of Field Crops, Batı Akdeniz Agricultural Research Institute, 07100 Antalya, Türkiye; sekip.erdal@tarimorman.gov.tr (Ş.E.); mehmet.pamukcu@tarimorman.gov.tr (M.P.); mehmet.kocturk@tarimorman.gov.tr (M.K.); 3 Department of Agricultural Structures and Irrigation, Faculty of Agriculture, Akdeniz University, 07058 Antalya, Türkiye; cihankaraca@akdeniz.edu.tr (C.K.); dbuyuktas@akdeniz.edu.tr (D.B.); 4 Department of Economy, Batı Akdeniz Agricultural Research Institute, 07100 Antalya, Türkiye; serifegulden.yilmaz@tarimorman.gov.tr; 5 Department of Agricultural Structures and Irrigation, Faculty of Agriculture, Ondokuz Mayıs University, 55270 Samsun, Türkiye; bcemek@omu.edu.tr

**Keywords:** subsurface drip irrigation, silage maize, deficit irrigation, nitrogen fertilization, water productivity, economic analysis

## Abstract

Excessive or poorly timed nitrogen (N) fertilization can increase nitrate leaching and associated environmental risks. This two-year field study evaluated the combined effects of three irrigation regimes and four N rates on the biomass yield, water productivity, and economic performance of silage maize under subsurface drip irrigation (SDI). The irrigation treatments were full irrigation (I_1_), 50% of I_1_ (deficit irrigation, I_2_), and 120% of I_1_ (excess irrigation, I_3_); the N treatments were 0 (N_0_), 140 (N_1_), 210 (N_2_), and 280 kg N ha^−1^ (N_3_). The experiment was established in a randomized complete block split-plot design with three replications during the 2022 and 2023 growing seasons. Seasonal irrigation ranged from 198 to 471 mm in 2022 and from 214 to 470 mm in 2023. The N_1_I_2_ treatment consistently produced the highest green forage yield (109,520 and 112,330 kg ha^−1^ in 2022 and 2023, respectively) while using approximately half the irrigation water applied to I_1_. It also produced the highest reported water productivity and irrigation water productivity, with values of 42.4 and 55.3 kg m^−3^ in 2022 and 44.4 and 52.5 kg m^−3^ in 2023, respectively. According to the partial-budget analysis, N_1_I_2_ generated the highest net profit (USD 31,941.83 ha^−1^ in 2022 and USD 28,500.08 ha^−1^ in 2023) and relative profit ratios of 4.17 and 11.48, respectively. Under the soil, climate, and price conditions of this study, applying 140 kg N ha^−1^ with deficit irrigation at 50% of the full-irrigation amount provided the best balance among biomass production, irrigation water use, and economic return.

## 1. Introduction

Maize (*Zea mays* L.) is one of the world’s most important cereal crops and is cultivated across diverse agroecological zones for grain, forage, and industrial uses. Silage maize is particularly valuable as ruminant feed because of its high biomass production, fermentable carbohydrate content, and digestibility. As demand for animal-derived products increases, improving the resource efficiency of silage maize production has become an important component of sustainable agricultural intensification [1,2].

Maize production depends strongly on water and nitrogen (N), which support cell expansion, chlorophyll formation, photosynthesis, and biomass accumulation. Nitrogen deficiency restricts leaf-area development and radiation interception, whereas excessive N application can increase lodging, delay maturity, reduce N-use efficiency, and contribute to environmental losses [3,4].

Water scarcity further constrains sustainable crop production. Agriculture accounts for approximately 70% of global freshwater withdrawals, and climate change is expected to increase drought risk and atmospheric evaporative demand in many maize-growing regions. Under water-limited conditions, inadequate irrigation management can reduce yield and alter nutrient availability. Because nitrate delivery to roots is strongly influenced by transpiration-driven mass flow, soil-water availability also affects plant N uptake [5,6].

Water stress affects maize growth, development, and yield; however, the yield attainable from a given water allocation depends on more than the total seasonal irrigation amount. Irrigation timing, soil-water stored in the root zone at crop establishment, and effective rainfall during the growing season also influence crop response [7,8].

Previous studies have shown that silage maize yield is sensitive to both irrigation timing and irrigation level [9,10,11,12,13]. Water deficits during tasseling, silking, pollination, and grain filling are especially damaging because these reproductive stages coincide with high crop evapotranspiration (ETc). Yield reductions have therefore been related to decreases in ETc and transpiration, and stage-specific yield-ETc relationships have been proposed [14,15].

Subsurface drip irrigation (SDI) is a precision-irrigation technology that can improve water productivity when it is properly designed and managed. Locating laterals below the soil surface reduces direct soil evaporation, facilitates uniform water delivery within the root zone, and enables precise fertigation. Compared with less precisely managed surface or sprinkler systems, SDI can reduce non-beneficial water losses; however, excessive irrigation can still cause deep percolation and nutrient leaching.

The response of maize to irrigation and N fertilization is interactive. Adequate N promotes canopy development and biomass accumulation, whereas excessive N under wet soil conditions may increase vegetative growth without a proportional increase in harvestable biomass. The optimum N rate therefore depends on irrigation method, soil properties, climate, cultivar, and management. Most previous studies have emphasized grain maize or conventional irrigation, and fewer have evaluated silage maize under SDI.

Silage maize differs from grain maize in harvest stage, biomass partitioning, and nutrient accumulation. Because nearly all aboveground biomass is harvested, often near the milk-to-dough stage, its seasonal N demand and water-use pattern may differ from those of grain maize. Determining crop-specific N requirements under SDI is therefore necessary to avoid both insufficient and excessive fertilization.

Increasing competition for water has also led to measures such as metering agricultural withdrawals, restricting new wells in water-stressed areas, and promoting region-specific cropping patterns. Under these conditions, reliable estimates of yield response to a limited water allocation are essential for water-intensive crops such as maize [16].

Although SDI has been investigated in Türkiye, region-specific evidence on the technical and economic performance of N fertigation for silage maize remains limited. Differences in soil texture, climate, cultivar, and management prevent a single N recommendation from being applied universally. Accordingly, this study evaluated the combined effects of three irrigation levels and four N rates on green forage yield, dry matter yield, water productivity, irrigation water productivity, and economic performance of silage maize under SDI. The objective was to identify a management combination that balanced biomass production, resource-use efficiency, and economic return under Mediterranean conditions.

## 2. Materials and Methods

### 2.1. Study Area and Climate

The experiment was conducted in 2022 and 2023 at the Batı Akdeniz Agricultural Research Institute (BATEM) in Antalya, Türkiye (36°56′29″ N, 30°53′04″ E; 11 m above sea level). Long-term monthly climate data (1930–2023) and data for the two experimental growing seasons are presented in Table 1. The site has a Mediterranean climate characterized by hot, dry summers and mild, wet winters. Mean growing-season air temperature was 25.9 °C in 2022 and 26.3 °C in 2023; corresponding precipitation totals were 12.0 and 123.6 mm, and mean relative humidity values were 66.2% and 67.2%, respectively. Climate data were obtained from the meteorological station at the experimental site.

### 2.2. Soil and Irrigation Water Characteristics

Selected physical and chemical properties of the experimental soil are presented in Table 2. Gravimetric field capacity ranged from 23.1% to 23.5%, and the permanent wilting point ranged from 10.8% to 11.7% across the 0–120 cm soil profile.

Selected chemical properties of the irrigation water are presented in Table 3. The electrical conductivity (EC) was 0.56 dS m^−1^ and the sodium adsorption ratio (SAR) was 0.34. The water was classified as C2S1, indicating medium salinity and a low sodium hazard.

### 2.3. Agronomic Practices

The Pehlivan 07 silage maize cultivar, registered by BATEM, was used. Registration records report a plant height of 300–310 cm, a dry matter content of 31.5%, and a silage yield of 73.990 kg ha^−1^. Seeds were sown at a row spacing of 70 cm, an intra-row spacing of 15 cm, and a depth of 3–5 cm. Sowing was performed mechanically on 18 May 2022 and 16 May 2023.

### 2.4. Irrigation System and Installation

The SDI control unit consisted of sand-and-gravel and disc filters, a pressure gauge, a water meter, valves and fittings, a fertilizer tank, and a fertilizer-injection system. The polyethylene mainline, manifolds, and laterals were installed below ground. Laterals were buried 40 cm below the soil surface and spaced 70 cm apart. Emitters were spaced 30 cm apart and had a nominal discharge of 2.1 L h^−1^.

### 2.5. Experimental Design and Treatments

**Figure 1:**
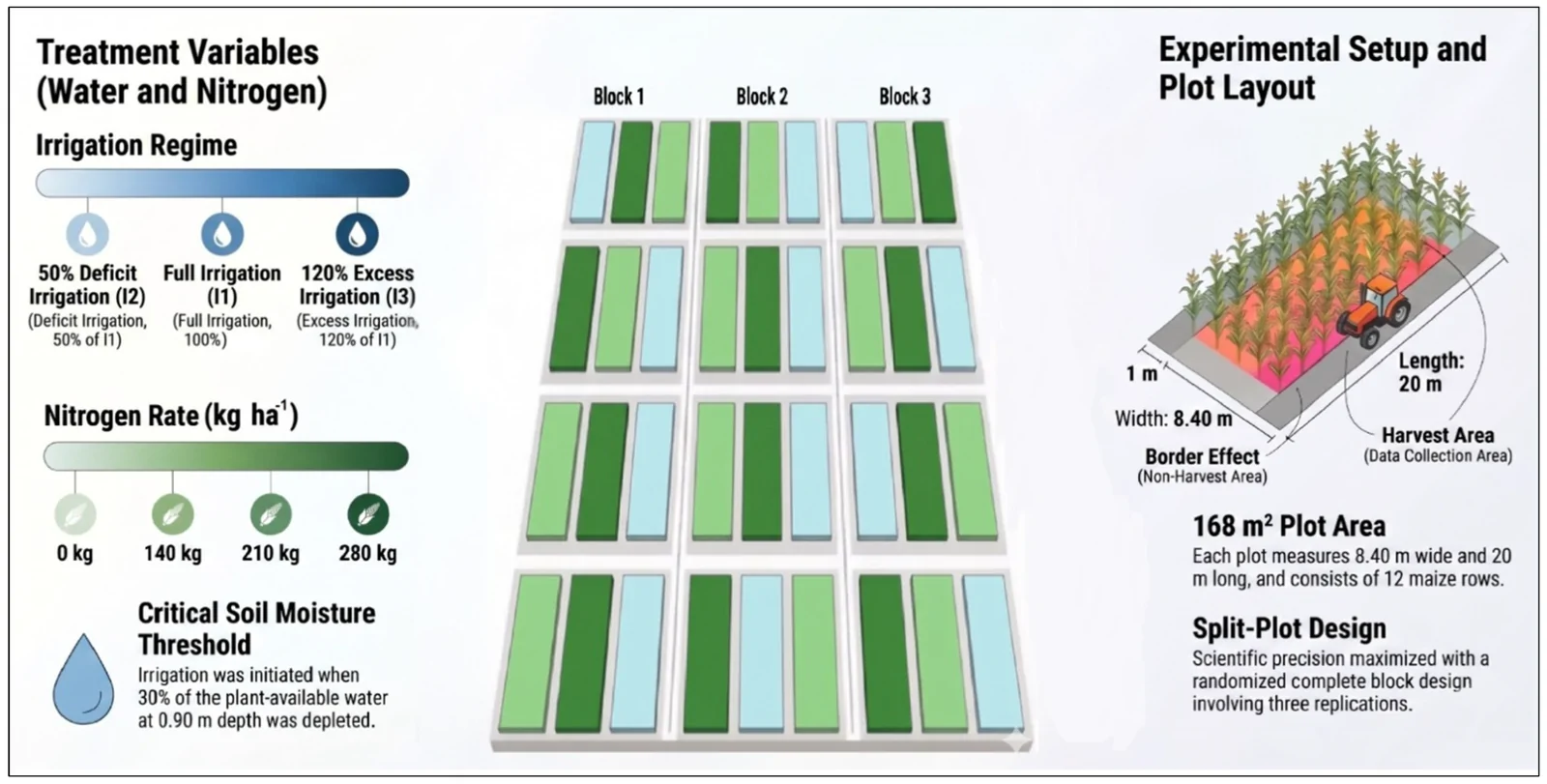
Layout and field implementation of the irrigation and nitrogen treatments.

The experiment included four N rates (N_0_: 0; N_1_: 140; N_2_: 210; and N_3_: 280 kg N ha^−1^) and three irrigation levels. I_1_ was the full-irrigation treatment, I_2_ received 50% of the water applied to I_1_, and I_3_ received 120% of the I_1_ amount. Treatment irrigation began when 30% of the available soil-water in the 0–0.90 m profile of I_1_ had been depleted. The I_1_ irrigation depth was the amount required to restore the 0–0.90 m soil profile to field capacity; I_2_ and I_3_ then received 50% and 120% of that amount, respectively (Figure 1).

The two-year field experiment was arranged as a randomized complete block split-plot design with three replications. Irrigation treatments (I_1_, I_2_, and I_3_) were assigned to the main plots, and the four N rates (N_0_, N_1_, N_2_, and N_3_) were independently randomized to subplots within each irrigation main plot. Thus, each block contained three main plots, and each main plot was divided into four nitrogen subplots. Each subplot consisted of 12 rows, was 8.40 m wide and 20.0 m long, and covered 168 m^2^. A 2.0 m buffer separated adjacent plots and blocks. The harvest area excluded 1 m from each end and one border row on each side, leaving the central 10 rows for yield determination.

**Figure 2:**
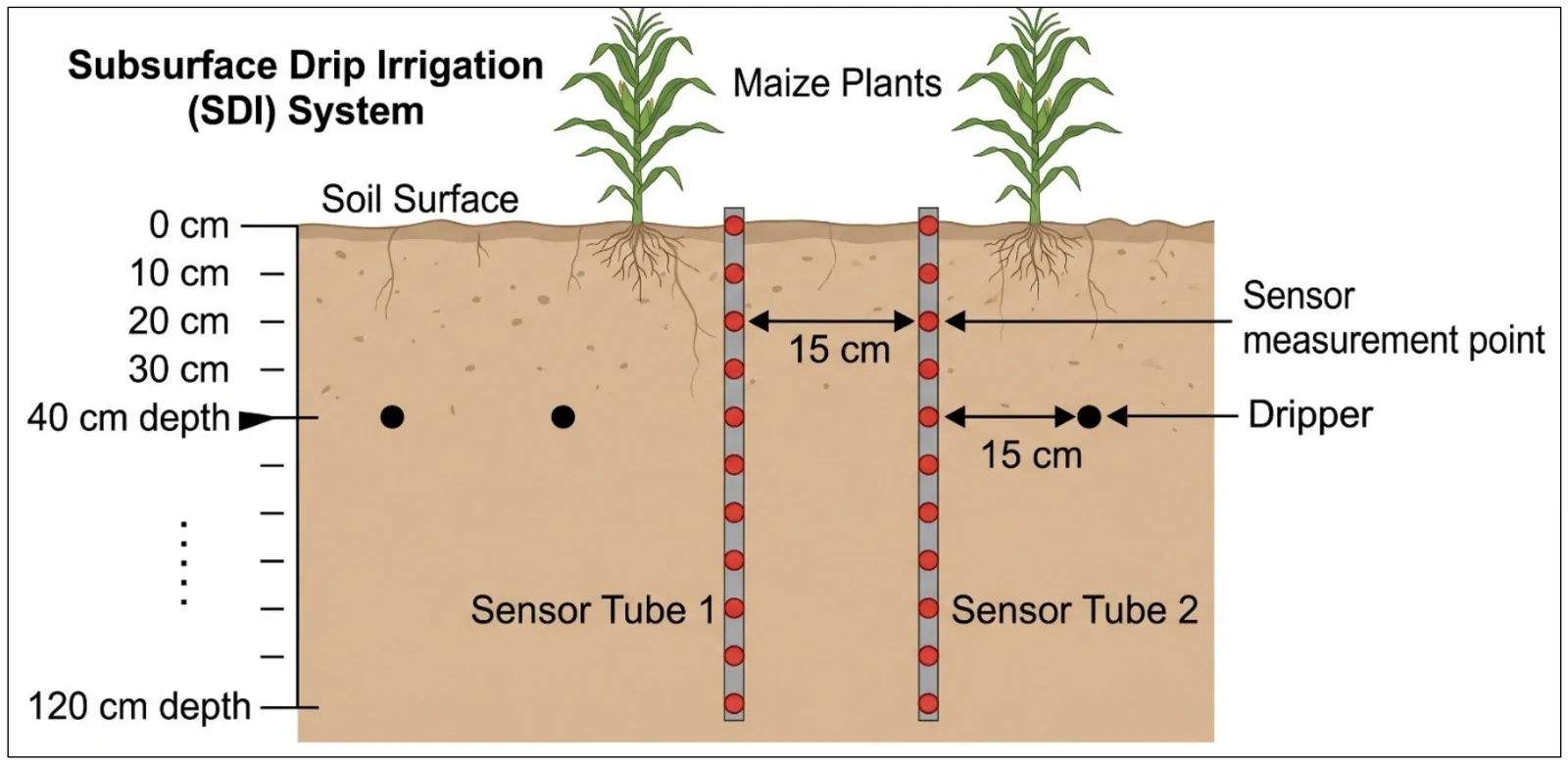
Positions of the soil-water probes, measurement zones, and drip emitters within the experimental plots.

Soil-water content was monitored using Drill & Drop (Sentek Sensor Technologies, 77 Magil Road Stepney, Adelaide, Australia) frequency-domain reflectometry (FDR) probes together with gravimetric sampling. Access probes were installed to a depth of 120 cm, and soil-water content was recorded at 10 cm depth intervals through the profile (Figure 2).

Urea (46% N) was used as the N source. One-fifth of the total N rate was applied at sowing, two-fifths at the six- to seven-leaf stage, and the remaining two-fifths at tasseling [17]. Fertilizer was applied with the irrigation water through a pressure-differential fertilizer tank [18].

### 2.6. Soil-Water Balance and Crop Evapotranspiration

Because SDI wetted only part of the soil surface, canopy cover (Pc) was used to scale irrigation and evapotranspiration calculations to the cropped area. A minimum Pc of 35% was used at the beginning of treatment irrigation; thereafter, measured Pc values were used. Irrigation depths for I_2_ and I_3_ were calculated as fixed proportions of the I_1_ depth.

For each irrigation treatment, the net irrigation depth for I_1_, which restored the allowable soil-water depletion to field capacity, was calculated using Equation (1). Treatments I_2_ and I_3_ received 50% and 120% of this amount, respectively.

(1) $$dn=\frac{\left(FC-WP\right)}{100}\times Ry\times D\times Pc\times \gamma t$$

In Equation (1), dn is the net irrigation depth (mm), FC is field-capacity water content (%), WP is permanent wilting-point water content (%), Ry is the allowable fraction of available soil-water depleted (%), D is effective root depth (mm), Pc is canopy cover (%), and γt is soil bulk density (g cm^−3^).

Crop evapotranspiration was calculated for the interval between successive soil-water measurements using the soil-water-balance approach (Equation (2)) [19,20,21]. The resulting ETc values were then adjusted using the measured Pc for each treatment.

(2) ETc=I+P±∆S−DP−RO

In Equation (2), ETc is crop evapotranspiration (mm), I is irrigation (mm), P is precipitation (mm), ΔS is the change in soil-water storage within the effective root zone (mm), DP is deep percolation (mm), and RO is surface runoff (mm). Deep percolation was estimated by monitoring soil-water changes below the 0.90 m root zone, at depths of 0.90–1.20 m.

### 2.7. Water Productivity and Irrigation Water Productivity

Water productivity (WP) was defined as the ratio of marketable yield to actual crop evapotranspiration (Equation (3)) [22].

(3) $$WP=\frac{Yield}{ETa}$$

In Equation (3), WP is water productivity (kg m^−3^), Y is marketable yield (kg ha^−1^), and ETa is actual evapotranspiration (mm). Unit conversion must be applied when yield is expressed per hectare and water depth is expressed in millimeters.

Irrigation water productivity (IWP) was defined as the ratio of marketable yield to the seasonal irrigation depth (Equation (4)) [23].

(4) $$\mathrm{IWP}=\frac{Yield}{I}$$

In Equation (4), IWP is irrigation water productivity (kg m^−3^), Y is marketable yield (kg ha^−1^), and I is seasonal irrigation depth (mm). Unit conversion must be applied when yield is expressed per hectare and irrigation is expressed as a depth.

### 2.8. Canopy Cover (Pc)

Canopy cover was measured before each irrigation on the same five preselected plants. Pc was calculated by dividing the mean width of the shaded canopy, projected perpendicular to the row, by the 70 cm row spacing. A minimum Pc of 35% was used until the measured value exceeded 35%, after which the measured value was used [24,25].

### 2.9. Leaf Area Index

Leaf area index (LAI) was determined using Equation (5).

LAI = Total leaf area/Corresponding ground area(5)

Three plants were sampled from each subplot. Their total leaf area was measured with a LI-COR LI-3000A (LI-COR Biosciences, 4647 Superior Street, Lincoln, NE, USA) leaf-area meter. The corresponding ground area was calculated from the fixed row spacing (0.70 m) and intra-row plant spacing (0.15 m), giving 0.105 m^2^ plant^−1^ and 0.315 m^2^ for the three sampled plants. LAI was calculated as total leaf area (m^2^) divided by the corresponding ground area (m^2^); therefore, LAI is dimensionless (m^2^ m^−2^). This calculation follows the plant-spacing approach described by Köksal et al. [26].

### 2.10. Green Forage Yield

All plants within the designated harvest area were cut and weighed. Plot-level fresh biomass was converted to green forage yield on a hectare basis (kg ha^−1^).

### 2.11. Dry Matter Yield

Three plants were selected randomly from each subplot and weighed fresh. Samples were oven-dried at 70 °C for 48 h and weighed again. Dry matter concentration was calculated as dry mass divided by fresh mass, and dry matter yield was obtained by multiplying this concentration by green forage yield.

### 2.12. Statistical Analysis

Data collected during the 2022 and 2023 growing seasons were subjected to a combined analysis of variance (ANOVA). The experiment was conducted as a randomized complete block split-plot design with three replications in each year. Irrigation level (IL), consisting of three levels (I_1_, I_2_, and I_3_), was assigned to the main plots, while nitrogen dose (ND), consisting of four levels (N_0_, N_1_, N_2_, and N_3_), was randomized to the subplots within each irrigation main plot. Thus, each block contained three irrigation main plots, each of which was divided into four nitrogen subplots. The combined analysis across years was performed according to the following statistical model:

(6) $$Y_{ijkl}=\mu +Y_{i}+B_{j\left(i\right)}+N_{k}+{\left(Y\times N\right)}_{ik}+e_{ijk}^{\left(a\right)}+I_{l}+{\left(Y\times I\right)}_{il}+{\left(N\times I\right)}_{kl}+{\left(Y\times N\times I\right)}_{ikl}+e_{ijkl}^{\left(b\right)}$$

where $Y_{ijkl}$ represents the observed response; μ is the overall mean; $Y_{i}$ is the effect of the ith year; $B_{j\left(i\right)}$ is the effect of the jth block nested within the ith year; $N_{k}$ is the effect of the kth nitrogen dose; $I_{l}$ is the effect of the lth irrigation level; and Y×N, Y×I, N×I, and Y×N×I represent the corresponding two- and three-way interactions. The term $e_{ijk}^{\left(a\right)}$ represents the main-plot error associated with the randomization of irrigation treatments within blocks, whereas $e_{ijkl}^{\left(b\right)}$ represents the subplot residual error associated with nitrogen treatments. The combined analysis across years was performed using a split-plot ANOVA model with year and block-within-year effects. Irrigation and its interaction with year were evaluated against the appropriate main-plot error, whereas nitrogen and interactions involving nitrogen were evaluated against the subplot residual error. Because irrigation had three levels, its treatment effect has 2 degrees of freedom. Nitrogen had four levels and therefore 3 degrees of freedom; the irrigation × nitrogen interaction had 6 degrees of freedom. Statistical significance was assessed at *p* < 0.05. The complete ANOVA results for green forage yield, dry matter yield, water productivity, and irrigation water productivity are provided in Supplementary Table S1.

### 2.13. Economic Analysis

A partial-budget approach was used to compare the additional benefits and costs associated with the irrigation and N treatments [27]. Gross production value, variable cost, total production cost, gross profit, net profit, production cost per kilogram, and relative profit were calculated for each treatment and year. The selling prices used in the analysis were USD 0.36 kg^−1^ in 2022 and USD 0.28 kg^−1^ in 2023. The variable and total production costs were retained as the basis of the economic comparison, and the treatment-related irrigation and N costs were included in the variable-cost calculations. All monetary calculations were first performed in Turkish Lira for each year and then converted to USD using the corresponding annual exchange rate (TRY 16.59 USD^−1^ in 2022 and TRY 24.11 USD^−1^ in 2023). Thus, exchange-rate differences between years were not pooled into a single conversion rate.

## 3. Results and Discussion

### 3.1. Irrigation and Crop Evapotranspiration

**Figure 3:**
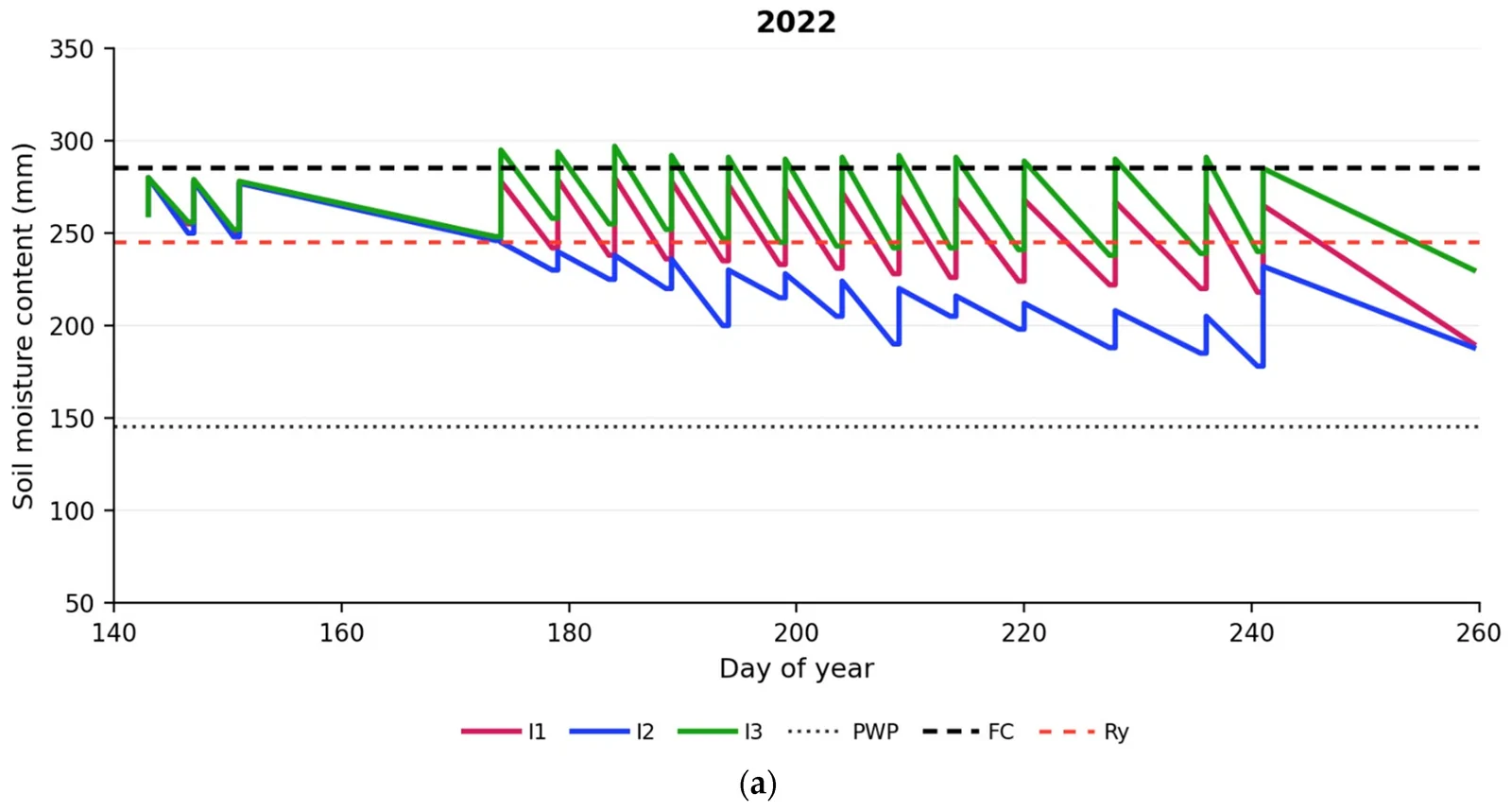

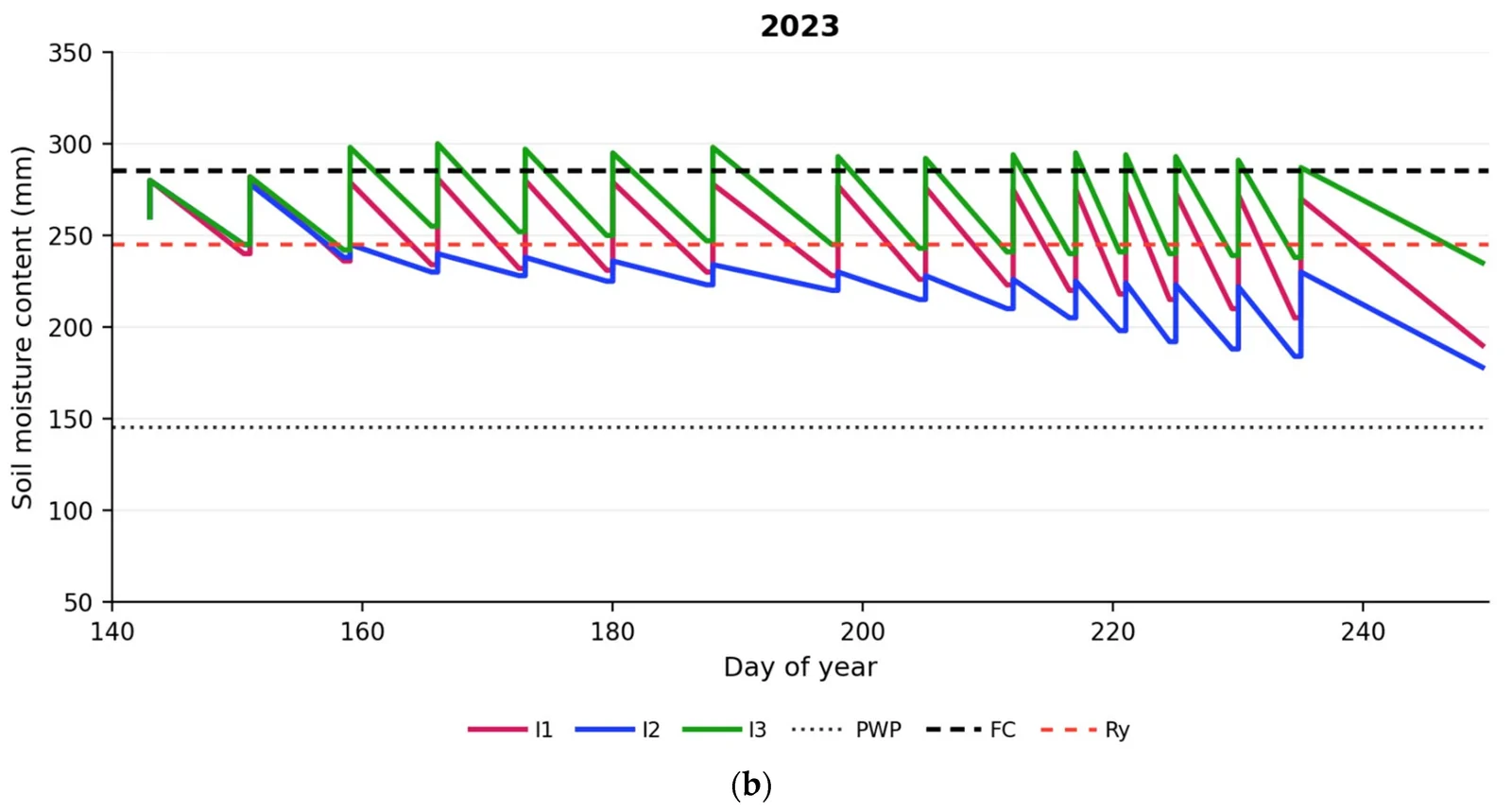
Root-zone soil-water dynamics under the irrigation treatments in (**a**) 2022 and (**b**) 2023. I_1_, full irrigation; I_2_, 50% of I_1_; I_3_, 120% of I_1_; PWP, permanent wilting point; FC, field capacity; Ry, allowable depletion.

All plots were irrigated uniformly until the five-leaf stage. Treatment irrigation was applied from 23 June to 29 August 2022 (13 irrigation events) and from 19 June to 29 August 2023 (12 events). Monthly and seasonal water-balance components are presented in Table 4, and temporal changes in root-zone soil-water are shown in Figure 3 for 2022 and 2023.

### 3.2. Canopy Cover

**Figure 4:**
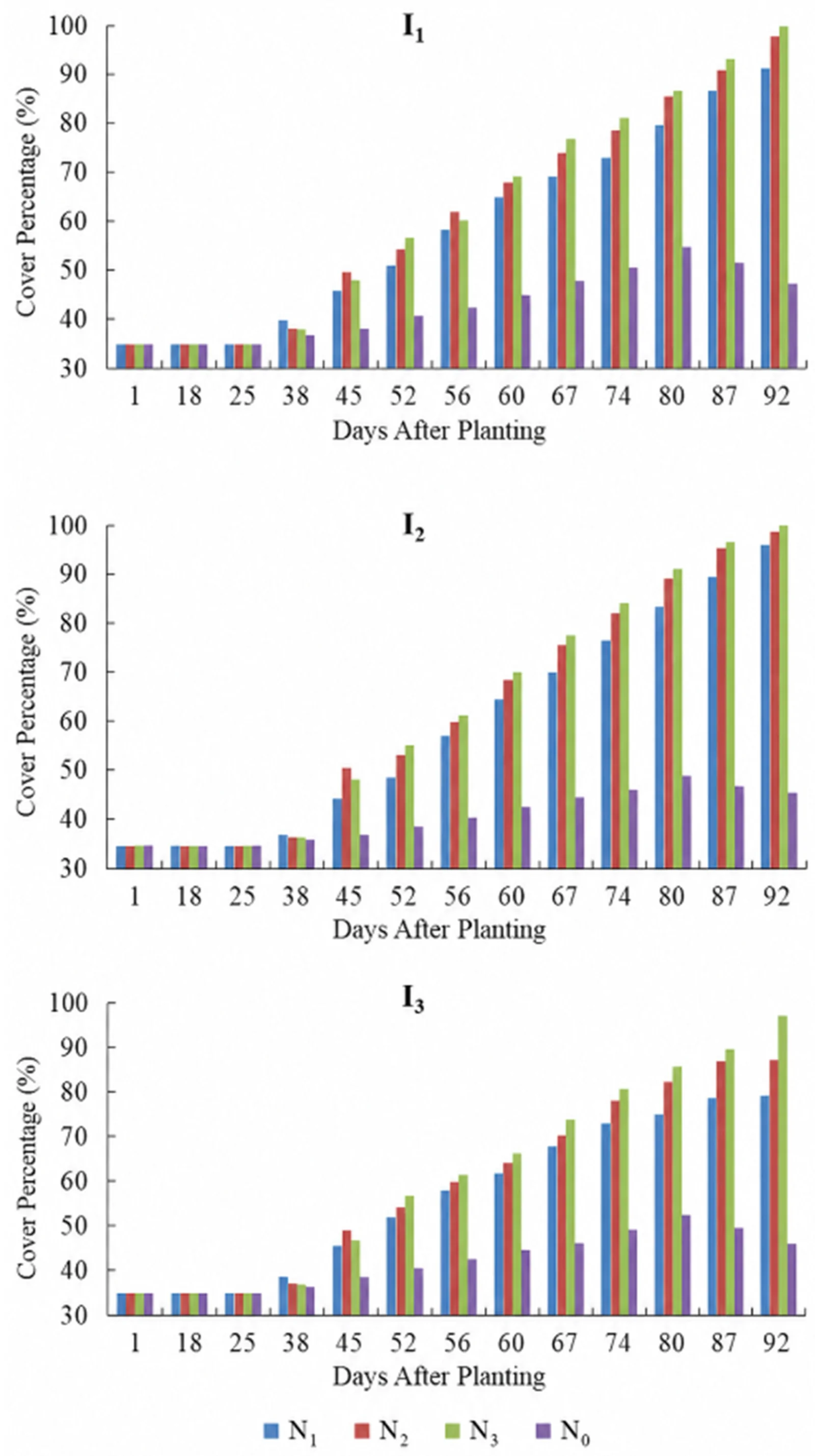
Seasonal variation in canopy cover under the irrigation and nitrogen treatments in 2022. N_0_, 0; N_1_, 140; N_2_, 210; and N_3_, 280 kg N ha^−1^; I_1_, full irrigation; I_2_, 50% of I_1_; I_3_, 120% of I_1_.

**Figure 5:**
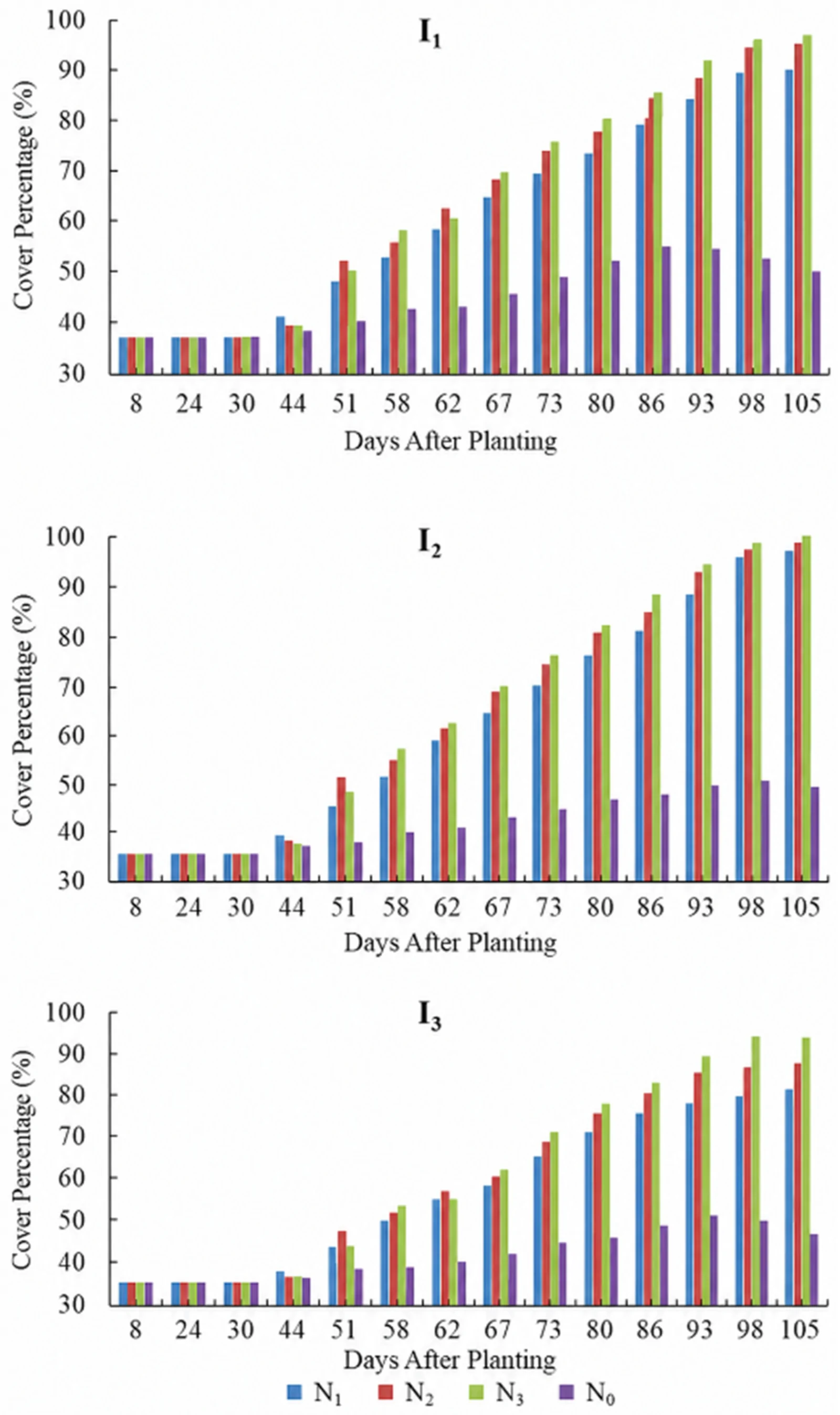
Seasonal variation in canopy cover under the irrigation and nitrogen treatments in 2023. N_0_, 0; N_1_, 140; N_2_, 210; and N_3_, 280 kg N ha^−1^; I_1_, full irrigation; I_2_, 50% of I_1_; I_3_, 120% of I_1_.

Canopy cover was set to 35% for irrigation calculations until measured Pc exceeded 35%; measured values were then used [24]. Pc was determined before each irrigation from the same five plants in each treatment. The seasonal patterns are shown in Figure 4 and Figure 5. Canopy development was slower in 2022 than in 2023, which may reflect the lower air and soil temperatures during May and June 2022. As canopy cover increases, a larger proportion of evapotranspiration is attributable to transpiration and a smaller proportion to direct soil evaporation. This shift influences both irrigation calculations and water productivity [28].

Ramachandiran and Pazhanivelan [29] reported canopy cover values of approximately 90% for maize under favorable growing conditions and observed a positive relationship between evapotranspiration and canopy development.

Greater canopy development generally increases crop water use by increasing transpiring leaf area [30,31]. It can also contribute to biomass production through greater light interception. These relationships should nevertheless be interpreted together with soil-water availability and plant N status.

### 3.3. Leaf Area Index

**Figure 6:**
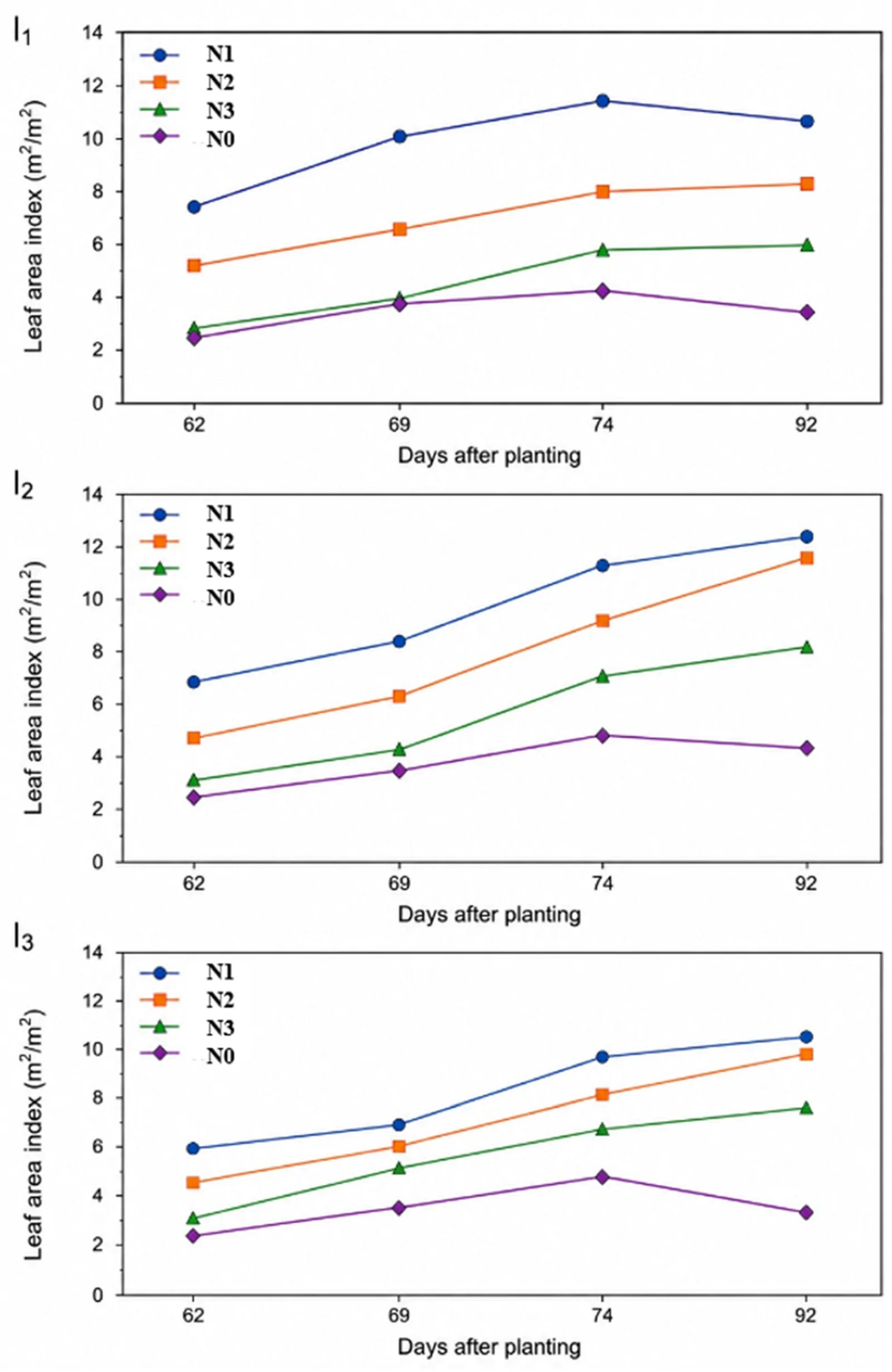
Seasonal variation in leaf area index under the irrigation and nitrogen treatments during the 2022 growing season. N_0_, 0; N_1_, 140; N_2_, 210; and N_3_, 280 kg N ha^−1^; I_1_, full irrigation; I_2_, 50% of I_1_; I_3_, 120% of I_1_.

**Figure 7:**
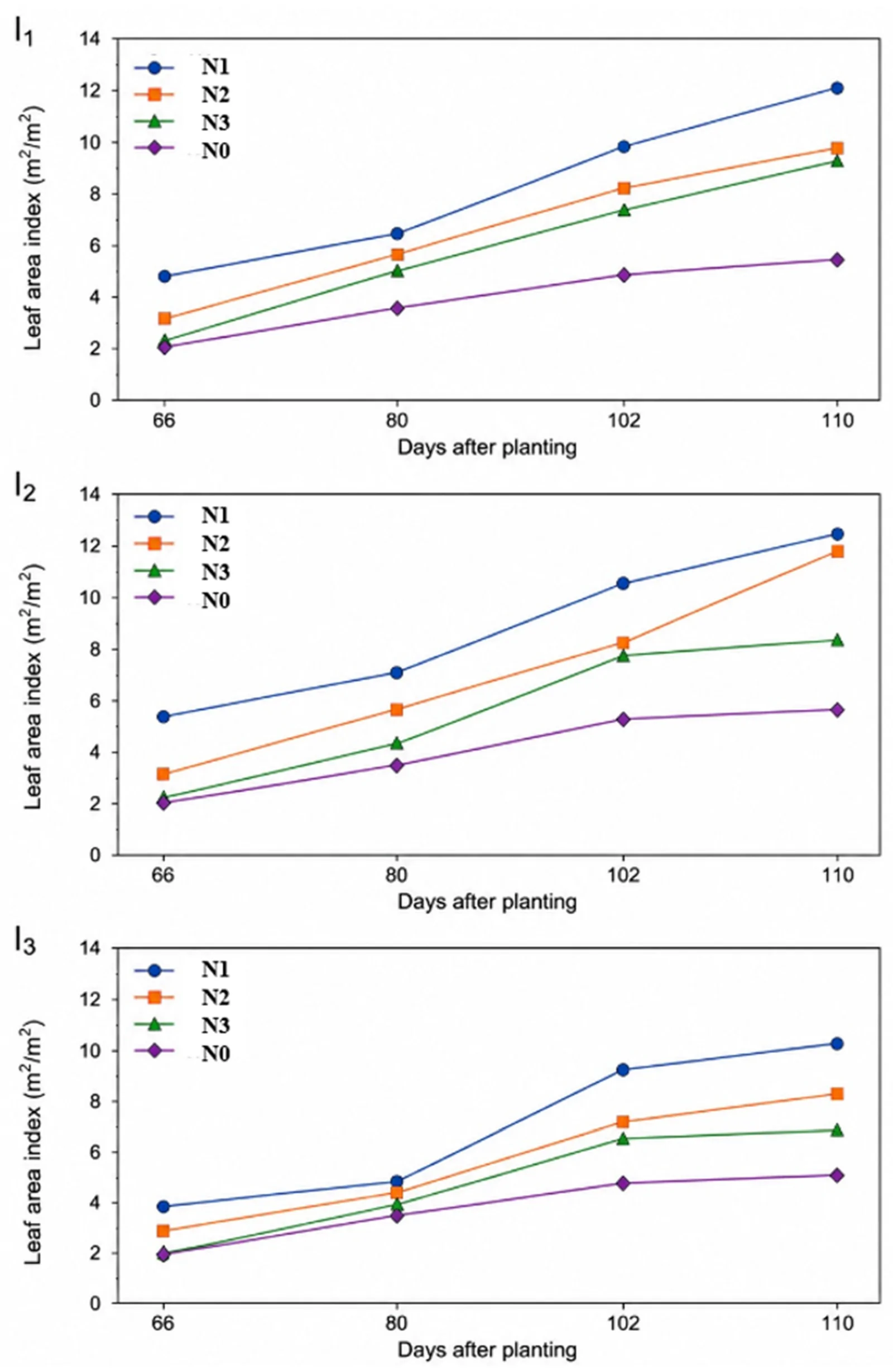
Seasonal variation in leaf area index under the irrigation and nitrogen treatments during the 2023 growing season. N_0_, 0; N_1_, 140; N_2_, 210; and N_3_, 280 kg N ha^−1^; I_1_, full irrigation; I_2_, 50% of I_1_; I_3_, 120% of I_1_.

Seasonal changes in LAI under the irrigation and N treatments are shown in Figure 6 and Figure 7. In both years, LAI increased from early vegetative growth to midseason and then declined as leaves senesced. The maximum LAI was observed under I_2_, whereas I_3_ generally produced the lowest maximum LAI. Maximum LAI values in 2022 were 11.6, 12.4, and 10.6 m^2^ m^−2^ for I_1_, I_2_, and I_3_, respectively; the corresponding values in 2023 were 12.1, 12.4, and 10.25 m^2^ m^−2^. The largest LAI values were generally associated with N_1_, and LAI did not increase consistently as the N rate increased. Deep percolation occurred under I_3_ (Table 4), indicating water movement below the monitored root-zone balance. However, because nitrate concentrations in soil- or drainage-water were not measured, nitrate leaching cannot be confirmed from deep percolation alone. Adequate soil-water supports cell division, leaf expansion, and canopy development, whereas water stress restricts leaf production and can accelerate senescence [32,33]. Across the two seasons, the maximum observed LAI values were 12.1 m^2^ m^−2^ for I_1_, 12.4 m^2^ m^−2^ for I_2_, and 10.6 m^2^ m^−2^ for I_3_. Zhu et al. [34] reported optimum LAI values of 10.93 and 11.12 m^2^ m^−2^ under irrigation levels slightly above 100% ETc and N rates of approximately 322–334 kg ha^−1^. Irmak et al. [35] reported mean maize LAI values of 6.40 and 6.03 m^2^ m^−2^ under different irrigation systems, whereas Liu et al. [36] reported maximum values near 5.0–5.3 m^2^ m^−2^. The comparatively high LAI values observed here may reflect differences in cultivar, plant density, environment, and measurement method.

### 3.4. Green Forage and Dry Matter Yields

Green forage and dry matter yields and Duncan groupings for the years 2022 and 2023 are presented in Table 5. Among the fertilized treatments, N_1_I_2_ produced the highest green forage yield in both 2022 (109,520 kg ha^−1^) and 2023 (112,330 kg ha^−1^), followed by N_2_I_2_ (105,780 and 96,800 kg ha^−1^, respectively). Among fertilized treatments, N_1_I_3_ produced the lowest green forage yield in both years (61,030 and 57,800 kg ha^−1^). These results indicate that increasing irrigation above the full-irrigation level did not increase biomass yield and that the crop response depended on the interaction between irrigation and N rate. The ANOVA results, including degrees of freedom, F statistics, and *p* values, are provided in Supplementary Tables S1 and S2.

Reported green forage yields in the literature vary widely with cultivar and environment. Ergül [37] reported 67,950–102,360 kg ha^−1^ in Konya, Güçük and Baytekin [38] reported 90,260–92,850 kg ha^−1^ in Şanlıurfa, and Çelebi and Türk [39] reported 55,090–103,160 kg ha^−1^ in Isparta. Kiziloglu et al. [40] found that silage-maize yield declined as water deficit increased and reported a linear relationship between crop evapotranspiration and green forage yield. Sarımehmetoğlu [41] reported dry biomass yields of 16,000–20,000 kg ha^−1^ for fully irrigated maize cultivars under Çukurova conditions.

### 3.5. Water Productivity and Irrigation Water Productivity

The reported WP and IWP values are presented in Table 5. N_1_I_2_ produced the highest values in both years: WP was 42.4 kg m^−3^ in 2022 and 44.4 kg m^−3^ in 2023, whereas IWP was 55.3 and 52.5 kg m^−3^, respectively. The green forage yield did not increase proportionally with irrigation amount. Under I_3_, yield remained lower than under I_1_ or I_2_ for several N rates, suggesting that water application above the full-irrigation amount was not beneficial under the conditions of this study. The ANOVA results, including degrees of freedom, F statistics, and *p* values, are provided in Supplementary Tables S3 and S4. Duncan groupings are given in Table 5.

On the basis of the reported WP and IWP values, N_1_I_2_ was the most efficient treatment. The reported values are within or above the ranges cited for maize by Karimi and Gomrokchi [42], Djaman and Irmak [43], and Gheysari et al. [12]. However, direct comparisons require the same yield basis (fresh biomass, dry biomass, or grain) and consistent conversion of yield and water depth to kg m^−3^.

Several studies have reported increases in yield as irrigation approaches the full-irrigation level [40,44]. In the present study, however, the highest reported IWP occurred under deficit irrigation. Excess water under SDI may increase deep percolation and reduce nutrient availability, but the proposed role of nitrate leaching cannot be confirmed without measurements of soil mineral N or drainage-water nitrate. The results therefore suggest that moderate N and deficit irrigation can improve the economic and apparent water-use performance of SDI-grown silage maize. Previous studies have also shown that deficit irrigation can either increase or decrease WP and IWP depending on stress severity, timing, irrigation method, and the yield component used in the calculation [40,45,46,47,48].

### 3.6. Economic Analysis

Because silage maize is an annual crop, treatment profitability was evaluated using partial budgeting. The analysis compared the additional revenue and costs associated with each irrigation-N combination. Results for 2022 and 2023 are summarized in Table 6 and Supplementary Tables S5 and S6. Maintaining adequate root-zone water and supplying N at an appropriate rate are important for maximizing both biomass yield and farm-level return.

In 2022, N_1_I_2_ (140 kg N ha^−1^ with deficit irrigation) produced the highest net profit (USD 31,941.83 ha^−1^), followed by N_2_I_2_ (USD 27,983.79 ha^−1^) and N_1_I_1_ (USD 18,362.88 ha^−1^). In 2023, N_1_I_2_ again produced the highest net profit (USD 28,500.08 ha^−1^), followed by N_1_I_1_ (USD 20,794.14 ha^−1^) and N_2_I_2_ (USD 18,659.62 ha^−1^). The selling prices used were USD 0.36 kg^−1^ in 2022 and USD 0.28 kg^−1^ in 2023, and the exchange rates were TRY 16.59 per USD in 2022 and TRY 24.11 per USD in 2023. Thus, under the prices and costs used in the analysis, deficit irrigation combined with the lowest nonzero N rate was the most profitable treatment.

## 4. Conclusions

This two-year study showed that the agronomic and economic responses of silage maize to N depended strongly on the irrigation level under SDI. Among the tested combinations, applying 140 kg N ha^−1^ with irrigation equal to 50% of the full-irrigation amount (N_1_I_2_) produced the highest green forage yield in both years, the highest WP and IWP values reported in the study, and the greatest net profit. Relative to I_1_, I_2_ reduced seasonal irrigation by approximately 49% in 2022 and 47% in 2023. Applying water above the full-irrigation amount did not improve yield and resulted in measured deep percolation. However, the occurrence of deep percolation should not be interpreted as direct evidence of nitrate leaching because nitrate concentrations were not measured. These findings support moderate N application combined with deficit irrigation as a potentially effective strategy for silage maize under the soil and Mediterranean climatic conditions of the study. Nevertheless, validation across additional sites and seasons is required before broad recommendations are made.

## Figures and Tables

**Table 1:** Monthly climatic conditions during the 2022 and 2023 growing seasons and the long-term period (1930–2023).

	May	June	July	August	September
**2022**					
Mean Daily Air Temperature (°C)	21.9	26.1	29.5	28.0	24.2
Total precipitation (mm)	8.00	5.00	0.0	0.0	0.0
Mean relative humidity (%)	61.1	69.8	54.2	80.4	65.6
**2023**					
Mean Daily Air Temperature (°C)	20.68	25.28	31.01	28.83	26.15
Total precipitation (mm)	91.0	27.0	0.0	0.0	6.2
Mean relative humidity (%)	74.0	69.2	49.5	79.1	64.4
**Long-term (1930–2023)**					
Mean daily air temperature (°C)	20.6	25.3	28.6	28.4	25.3
Total precipitation (mm)	34.3	11	4.4	4.3	16.9

**Table 2:** Physical and chemical properties of the experimental soil.

Depth (cm)	Clay (%)	Silt (%)	Sand (%)	Texture Class	CaCO_3_ (%)	pH	EC (dS m^−1^)	Bulk Density (g cm^−3^)	Field Capacity (%)	Wilting Point (%)
0–30	32	44	24	CL	25.6	8.3	0.10	1.31	23.5	10.8
30–60	28	48	24	CL	24.8	8.3	0.11	1.38	23.4	11.1
60–90	24	40	36	L	23.7	8.4	0.16	1.43	23.1	11.7
90–120	26	48	26	L	23.9	8.3	0.15	1.41	23.2	10.8

**Table 3:** Selected chemical properties of the irrigation water.

pH	EC (dS m^−1^)	Cations (meq L^−1^)				Anions (meq L^−1^)				Class
		Na^+^	K^+^	Ca^2+^	Mg^2+^	CO_3_^2−^	HCO_3_^−^	Cl^−^	SO_4_^2−^	
7.30	0.56	0.49	0.05	4.23	1.85	-	5.03	0.53	1.06	C**_2_**S**_1_**

**Table 4:** Monthly soil-water balance components for each irrigation treatment in 2022 and 2023 (mm).

Treatment	Month	2022					2023				
		I	P	∆S	DP	ET	I	P	∆S	DP	ET
I_1_	May	40	8	−24.9	0	23.1	10	91	−2.2	0	98.8
	June	115	5	30.1	0	150.1	105	27	7.8	0	139.8
	July	131	0	5.6	0	136.6	131	0	−12.0	0	119.0
	August	101	0	−3.2	0	97.8	157	0	−3.0	0	154.0
	Total	387	13	7.5	0	407.5	403	118	−9.4	0	511.6
I_2_	May	40	8	−24.9	0	21.3	10	91	−12.3	0	88.7
	June	63	5	40.6	0	108.6	53	27	−27.3	0	52.7
	July	58	0	19.9	0	77.9	67	0	−33.3	0	33.7
	August	37	0	11.8	0	48.8	84	0	−6.2	0	77.8
	Total	198	13	47.4	0	258.4	214	118	−79.1	0	252.9
I_3_	May	40	8	−24.9	0	28.1	10	91	5.4	0	106.4
	June	149	5	44.6	13	209.6	137	27	−10.1	17	170.9
	July	158	0	28.4	18	202.4	158	0	−19.9	22	160.1
	August	124	0	29.6	10	162.6	165	0	−5.2	14	173.8
	Total	471	13	77.7	41	602.7	470	118	−29.8	53	611.2

I_1_, full irrigation triggered when 30% of the available soil-water in the 0–0.90 m profile was depleted; I_2_, deficit irrigation receiving 50% of the I_1_ amount; I_3_, excess irrigation receiving 120% of the I_1_ amount; I, irrigation; P, precipitation; ΔS, change in soil-water storage; DP, deep percolation; ET, crop evapotranspiration.

**Table 5:** Green forage yield, dry matter yield, water productivity, and irrigation water productivity under the irrigation and nitrogen treatments.

Treatment		GFY (kg ha^−1^)		Mean	DMY (kg ha^−1^)		Mean	WP (kg m^−3^)		Mean	IWP (kg m^−3^)		Mean
Irrigation	Nitrogen	2022	2023	GFY	2022	2023	DMY	2022	2023	WP	2022	2023	IWP
I_1_	N_0_	27,650	29,500	28,575 k	10,050	10,510	10,280 ef	6.8	5.8	6.3 ı	7.1	7.3	7.2 ı
	N_1_	72,100	84,420	78,260 g	31,550	35,100	33,325 bd	17.7	16.5	17.1 f	18.6	20.9	19.8 ef
	N_2_	83,500	86,700	85,100 e	33,280	38,860	36,070 bc	20.5	16.9	18.7 e	21.6	21.5	21.5 de
	N_3_	92,450	93,100	92,775 c	36,600	39,910	38,255 bc	22.7	18.2	20.4 d	23.9	23.1	23.5 d
I_2_	N_0_	34,500	36,700	35,600 j	10,085	10,050	10,068 ef	13.4	14.5	13.9 g	17.4	17.1	17.3 fg
	N_1_	109,520	112,330	110,925 a	54,690	63,360	59,025 a	42.4	44.4	43.4 a	55.3	52.5	53.9 a
	N_2_	105,780	96,800	101,290 b	40,410	41,610	41,010 b	40.9	38.3	39.6 b	53.4	45.2	49.3 b
	N_3_	89,970	78,200	84,085 f	37,870	23,970	30,920 bc	34.8	30.9	32.9 c	4.54	36.5	41.0 c
I_3_	N_0_	26,400	24,800	25,600 L	8880	9200	9040 f	4.4	4.1	4.2 j	5.6	5.3	5.4 ı
	N_1_	61,030	57,800	59,415 ı	18,120	14,920	16,520 bd	10.1	9.5	9.8 h	13.0	12.3	12.6 h
	N_2_	75,810	76,200	76,005 h	21,250	22,650	21,950 de	12.6	12.5	12.5 g	16.1	16.2	16.2 g
	N_3_	85,230	87,530	86,380 d	29,140	22,800	25,970 cd	14.1	14.3	14.2 g	18.1	18.6	18.4 fg

Within a column, means followed by different letters differ significantly at *p* < 0.05. N_0_, 0; N_1_, 140; N_2_, 210; and N_3_, 280 kg N ha^−1^; I_1_, full irrigation; I_2_, 50% of I_1_; I_3_, 120% of I_1_; GFY, green forage yield; DMY, dry matter yield; WP, water productivity; IWP, irrigation water productivity.

**Table 6:** Partial-budget analysis of the irrigation and nitrogen treatments in 2022 and 2023.

	Treatments											
	N_1_I_1_	N_1_I_2_	N_1_I_3_	N_2_I_1_	N_2_I_2_	N_2_I_3_	N_3_I_1_	N_3_I_2_	N_3_I_3_	N_0_I_1_	N_0_I_2_	N_0_I_3_
**2022**												
Variable costs (USD ha^−1^)	7669.06	7623.65	7750.86	10,258.95	10,224.36	10,286.16	13,903.95	13,853.91	13,926.53	5588.19	5542.79	5599.97
Total production costs (USD ha^−1^)	7713.07	7667.57	7795.01	10,307.64	10,272.99	10,334.90	13,959.23	13,909.10	13,981.86	5628.43	5582.95	5640.23
Green forage yield (kg ha^−1^)	72,100.00	109,520.00	61,030.00	83,500.00	105,780.00	75,810.00	92,450.00	89,970.00	85,230.00	27,650.00	34,500.00	29,500.00
Production cost (USD kg^−1^)	0.11	0.07	0.13	0.12	0.10	0.14	0.15	0.15	0.16	0.20	0.16	0.19
Selling price (USD kg^−1^)	0.36	0.36	0.36	0.36	0.36	0.36	0.36	0.36	0.36	0.36	0.36	0.36
Gross production value (USD ha^−1^)	26,075.95	39,609.40	22,072.33	30,198.92	38,256.78	27,417.72	33,435.80	32,538.88	30,824.59	10,000.00	12,477.40	10,669.08
Gross profit (USD ha^−1^)	18,406.88	31,985.75	14,321.47	19,939.96	28,032.42	17,131.56	19,531.86	18,684.97	16,898.06	4411.81	6934.61	5069.11
Net profit (USD ha^−1^)	18,362.88	31,941.83	14,277.32	19,891.27	27,983.79	17,082.82	19,476.58	18,629.78	16,842.74	4371.57	6894.45	5028.85
Relative profit ratio	2.38	4.17	1.83	1.93	2.72	1.65	1.40	1.34	1.20	0.78	1.23	0.89
**2023**												
Variable costs (USD ha^−1^)	2466.71	2458.89	5899.13	8145.42	8008.97	8202.92	10,890.27	10,741.30	10,945.17	4628.29	4481.92	4673.26
Total production costs (USD ha^−1^)	2490.52	2482.68	5927.20	8176.29	8039.68	8233.87	10,924.56	10,775.41	10,979.53	4654.78	4508.24	4699.81
Green forage yield (kg ha^−1^)	84,420.00	112,330.00	57,800.00	86,700.00	96,800.00	76,200.00	93,100.00	78,200.00	87,530.00	29,500.00	36,700.00	24,800.00
Production cost (USD kg^−1^)	0.03	0.02	0.10	0.09	0.08	0.11	0.12	0.14	0.13	0.16	0.12	0.19
Selling price (USD kg^−1^)	0.28	0.28	0.28	0.28	0.28	0.28	0.28	0.28	0.28	0.28	0.28	0.28
Gross production value (USD ha^−1^)	23,284.65	30,982.77	15,942.35	23,913.52	26,699.29	21,017.42	25,678.76	21,569.06	24,142.45	8136.67	10,122.56	6840.32
Gross profit (USD ha^−1^)	20,817.94	28,523.88	10,043.22	15,768.10	18,690.32	12,814.50	14,788.49	10,827.76	13,197.28	3508.38	5640.64	2167.05
Net profit (USD ha^−1^)	20,794.14	28,500.08	10,015.14	15,737.23	18,659.62	12,783.55	14,754.20	10,793.65	13,162.92	3481.88	5614.33	2140.50
Relative profit ratio	8.35	11.48	1.69	1.92	2.32	1.55	1.35	1.00	1.20	0.75	1.25	0.46

N_0_, 0; N_1_, 140; N_2_, 210; and N_3_, 280 kg N ha^−1^; I_1_, full irrigation; I_2_, 50% of I_1_; I_3_, 120% of I_1_. Monetary values are expressed in 2022 or 2023 USD, as applicable. Selling prices were USD 0.36 kg^−1^ in 2022 and USD 0.28 kg^−1^ in 2023. The exchange rates used were TRY 16.59 per USD in 2022 and TRY 24.11 per USD in 2023.

## Data Availability

The original contributions presented in this study are included in the article/Supplementary Materials. Further inquiries can be directed to the corresponding author.

## Supplementary Materials

The following supporting information can be downloaded at https://www.mdpi.com/article/10.3390/plants15172734/s1, Table S1: Results of variance analysis of green forage yield for research topics; Table S2: Results of variance analysis of dry matter yield for research topics; Table S3: Results of variance analysis of water productivity for research topics; Table S4: Results of variance analysis of irrigation water productivity for research topics; Table S5: Economic analysis of different irrigation levels and nitrogen rates in silage maize (2022 year); Table S6: Economic analysis of different irrigation levels and nitrogen rates in silage maize (2023 year).
