# Supplementary material for: Agronomic Response of Silage Maize to Varying Irrigation Water and Nitrogen Levels Under Subsurface Drip Irrigation: Implications for Yield and Water Use Productivity
Source: Plants (Basel). 2026 Sep 7;15(17):2734. doi: 10.3390/plants15172734 (PMC13567550; doi:10.3390/plants15172734)
Supplement: Supplementary file 1 [file plants-15-02734-s001.zip › plants-4400279-supplementary.pdf]

Table S1. Results of variance analysis of green forage yield for research topics

Sources of variation	df	SS	MS	F	P-value>F
Year (Y)	1	901,001	901,001	0,5671	0,4799
Block (B)	2	74521	37260,5	23,4512	0,0015
Nitrogen Dose (ND)	3	4,26E+08	1,42E+08	89435,16	<,0001*
Y*ND	3	1258262	419421	263,9769	<,0001*
B*ND	6	11514,6	1919,11	1,2079	0,4123
Y*B	2	4269,06	2134,53	1,3434	0,3295
Irrigation Level (IL)	2	5,31E+07	2,66E+07	11759,75	<,0001*
Y*IL	2	2081691	1040846	460,9078	<,0001*
IL*ND	6	5,24E+07	8731581	3866,523	<,0001*
Y*IL*ND	6	2885569	480928	212,9649	<,0001*
Y*B*ND&Random (error1)	6	9,53E+03	1588,85	0,7036	0,6489
Y*B*IL*ND&Random (error2)	32	72264	2258,251		
Corrected total	71	5,38E+08			<,0001*
CV (%)	1,6				

*: $p < 0.05$ and **; $p < 0.01$. ns; not important.

Table S2. Results of variance analysis of dry matter yield for research topics

**Sources of variation**	**df**	**SS**	**MS**	**F**	**P-value>F**
Year (Y)	1	294685	294685	0,3496	0,5759
Block (B)	2	1944751	972375	1,1536	0,3768
Nitrogen Dose (ND)	3	9,01E+07	3,00E+07	35,6391	0,0003*
Y*ND	3	5599197	1866399	2,2142	0,1872
B*ND	6	4513270	752212	0,8924	0,5532
Y*B	2	952918	476459	0,5652	0,5958
Irrigation Level (IL)	2	2,23E+07	1,12E+07	12,8336	<,0001*
Y*IL	2	8422547	4211273	4,842	0,0146*
IL*ND	6	1,63E+07	2718801	3,126	0,0159*
Y*IL*ND	6	2,24E+07	3734535	4,2938	0,0028*
Y*B*ND&Random (error1)	6	5,06E+06	842928	0,9692	0,4616
Y*B*IL*ND&Random (error2)	32	27831733	869742		
Corrected total	71	2,06E+08			<,0001*
CV (%)	0,33				

*: p < 0.05 and **; p < 0.01. ns; not important.

Table S3. Results of variance analysis of water productivity for research topics

Sources of variation	df	SS	MS	F	P-value>F
Year (Y)	1	0,00125	0,00125	0,0462	0,837
Block (B)	2	0,00842	0,00421	0,1556	0,8593
Nitrogen Dose (ND)	3	3,14E+01	1,05E+01	387,2115	<,0001*
Y*ND	3	0,11643	0,03881	1,434	0,3228
B*ND	6	0,06788	0,01131	0,418	0,8437
Y*B	2	0,02481	0,0124	0,4583	0,6528
Irrigation Level (IL)	2	5,79E+01	2,90E+01	4212,238	<,0001*
Y*IL	2	0,22416	0,11208	16,3041	<,0001*
IL*ND	6	9,15E+00	1,5249	221,8264	<,0001*
Y*IL*ND	6	2,30E-01	0,03833	5,5754	0,0005*
Y*B*ND&Random (error1)	6	1,62E-01	0,02706	3,9369	0,0047*
Y*B*IL*ND&Random (error2)	32	0,219978	0,00687		
Corrected total	71	9,96E+01			<,0001*
CV (%)	0,41				

*: $p < 0.05$ and **; $p < 0.01$. ns; not important.

Table S4. Results of variance analysis of irrigation water productivity for research topics

Sources of variation	df	SS	MS	F	P-value>F
Year (Y)	1	0,11681	0,11681	2,2148	0,1873
Block (B)	2	0,11253	0,05627	1,0669	0,4014
Nitrogen Dose (ND)	3	4,46E+01	1,49E+01	281,6795	<,0001*
Y*ND	3	0,69292	0,23097	4,3795	0,0589
B*ND	6	0,16251	0,02709	0,5136	0,7812
Y*B	2	0,04948	0,02474	0,4691	0,6467
Irrigation Level (IL)	2	9,56E+01	4,78E+01	1240,511	<,0001*
Y*IL	2	0,33325	0,16663	4,3255	0,0217*
IL*ND	6	1,64E+01	2,73656	71,0385	<,0001*
Y*IL*ND	6	4,19E-01	0,06979	1,8116	0,1282
Y*B*ND&Random (error1)	6	3,16E-01	0,05274	1,3691	0,2569
Y*B*IL*ND&Random (error2)	32	1,23271	0,03852		
Corrected total	71	1,60E+02			<,0001*
CV (%)	0,08				

*: p < 0.05 and **; p < 0.01. ns; not important.

Table S5. Economic analysis of different irrigation levels and nitrogen rates in silage maize (2022 year)

Variables	Unit	Amount		Expenses ($)	TREATMENTS (N_1: 140 kg N ha^{-1}, N_2: 210 kg N ha^{-1} , N_3: 280 kg N ha^{-1} and N_0: 0 (No nitrogen application), I_1: %100, I_2: %50 ve I_3: %120											
					N_1I_1	N_1I_2	N_1I_3	N_2I_1	N_2I_2	N_2I_3	N_3I_1	N_3I_2	N_3I_3	N_0I_1	N_0I_2	N_0I_3
**Inputs**																
Fertilizer expenses	kg ha^{-1}	N_1	140	13,50	1890	1890	1890	4253	4253	4253	7561	7561	7561	0	0	0
		N_2	210	20,25												
		N_3	280	27,00												
		N_0	0	0,00												
Seed expenses	kg ha^{-1}	25,0		18,08	4521	4521	4521	4521	4521	4521	4521	4521	4521	4521	4521	4521
Expenses of agricultural pesticides	kg ha^{-1}			11,39	113,92	113,92	113,92	113,92	113,92	113,92	113,92	113,92	113,92	113,92	113,92	113,92
Irrigation expenses	m³			0,02	81,6	41,8	99,4	81,6	41,8	99,4	81,6	41,8	99,4	81,6	41,8	99,4
Electricity used	h				72,0	61,2	50,5	7,6	72,0	61,2	50,5	7,6	72,0	61,2	50,5	7,6
Electricity expenses	kWh			0,13	9,4	8,0	66,1	1,0	9,4	8,0	6,6	1,0	9,4	8,0	6,6	1,0
**Input expenses**					6616,08	6574,79	6690,44	8970,52	8939,07	8995,26	12284,15	12238,67	12304,69	4724,37	4683,10	4735,08
**Labour**																
Plowing	ha	10,0		4,63	46,33	46,33	46,33	46,33	46,33	46,33	46,33	46,33	46,33	46,33	46,33	46,33
Rotary tiller	ha	10,0		4,03	40,30	40,30	40,30	40,30	40,30	40,30	40,30	40,30	40,30	40,30	40,30	40,30
Planting	ha	10,0		3,79	37,89	37,89	37,89	37,89	37,89	37,89	37,89	37,89	37,89	37,89	37,89	37,89
Inter-row cultivation	ha	10,0		3,67	36,68	36,68	36,68	36,68	36,68	36,68	36,68	36,68	36,68	36,68	36,68	36,68
Agricultural pest control	ha	10,0		3,32	33,22	33,22	33,22	33,22	33,22	33,22	33,22	33,22	33,22	33,22	33,22	33,22
Irrigation and fertilization labor	ha	10,0		3,55	35,48	35,48	35,48	35,48	35,48	35,48	35,48	35,48	35,48	35,48	35,48	35,48
Facility maintenance and repair	ha	10,0		3,55	35,48	35,48	35,48	35,48	35,48	35,48	35,48	35,48	35,48	35,48	35,48	35,48
Harvest	ha	10,0		9,04	90,42	90,42	90,42	90,42	90,42	90,42	90,42	90,42	90,42	90,42	90,42	90,42
**Labor expenses**					355,80	355,80	355,80	355,80	355,80	355,80	355,80	355,80	355,80	355,80	355,80	355,80
**Input and labor expenses**					6971,88	6930,59	7046,24	9326,32	9294,87	9351,06	12639,95	12594,47	12660,48	5080,17	5038,90	5090,88
Revolving fund interest					697,19	693,06	704,62	932,63	929,49	935,11	1264,00	1259,45	1266,05	508,02	503,89	509,09
**Variable Expenses**	$				7669,06	7623,65	7750,86	10258,95	10224,36	10286,16	13903,95	13853,91	13926,53	5588,19	5542,79	5599,97
Unexpected expenses					13,87	13,79	14,02	18,55	18,49	18,60	25,14	25,05	25,18	10,11	10,02	10,13
Field rent	ha	10,0			0	0	0	0	0	0	0	0	0	0	0	0
Depreciation allowance for irrigation systems	ha	1000-1800			30,14	30,14	30,14	30,14	30,14	30,14	30,14	30,14	30,14	30,14	30,14	30,14
**Fixed Expenses**					44,01	43,92	44,15	48,69	48,63	48,74	55,28	55,19	55,32	40,24	40,16	40,27
**Total Production Costs**	$				7713,07	7667,57	7795,01	10307,64	10272,99	10334,90	13959,23	13909,10	13981,86	5628,43	5582,95	5640,23
Green forage yield	kg ha^{-1}				72100	109520	61030	83500	105780	75810	92450	89970	85230	27650	34500	29500
Production cost	$ ha^{-1}				0,107	0,07	0,13	0,12	0,10	0,14	0,15	0,15	0,16	0,20	0,16	0,19
Selling price	$ ha^{-1}	10,0			0,36	0,36	0,36	0,36	0,36	0,36	0,36	0,36	0,36	0,36	0,36	0,36
Gross production value	$				26075,95	39609,40	22072,33	30198,92	38256,78	27417,72	33435,80	32538,88	30824,59	10000,00	12477,40	10669,08
Gross profit	$				18406,88	31985,75	14321,47	19939,96	28032,42	17131,56	19531,86	18684,97	16898,06	4411,81	6934,61	5069,11
Net profit	$				18362,88	31941,83	14277,32	19891,27	27983,79	17082,82	19476,58	18629,78	16842,74	4371,57	6894,45	5028,85
Relative profit	%				2,38	4,17	1,83	1,93	2,72	1,65	1,40	1,34	1,20	0,78	1,23	0,89

N_0: No nitrogen application, N_1: 140 kg N ha^{-1} nitrogen application; N_2: 210 kg N ha^{-1} nitrogen application; N_3: 280 kg N ha^{-1} nitrogen application, I_1:full irrigation; 100%, I_2:deficit irrigation; 50% of I_1, and I_3:excess irrigation, 120% of I_1. The USD value used in the economic analysis refers to the years 2022 and 2023, when the research was conducted. In 2022, 1 USD was equal to 16.59 Turkish Lira, and in 2023 it was equal to 24.11 Turkish Lira.

Table S6. Economic analysis of different irrigation levels and nitrogen rates in silage maize (2023 year)

Variables	Unit	Amount		Expenses ($)	TREATMENTS (N1: 140 kg N ha-1, N2: 210 kg N ha-1, N3: 280 kg N ha-1 and N0: 0 (No nitrogen application), I1: %100, I2: %50 ve I3: %120											
					N_1I_1	N_1I_2	N_1I_3	N_2I_1	N_2I_2	N_2I_3	N_3I_1	N_3I_2	N_3I_3	N_0I_1	N_0I_2	N_0I_3
**Inputs**																
Fertilizer expenses	kg ha^{-1}	N_1	140	10,09	1413	1413	1413	3179	3179	3179	5652	5652	5652	0	0	0
		N_2	210	15,14												
		N_3	280	20,18												
		N_0	0	0,00												
Seed expenses	kg ha^{-1}	25		13,77	344,26	344,26	3442,55	3442,55	3442,55	3442,55	3442,55	3442,55	3442,55	3442,55	3442,55	3442,55
Expenses of agricultural pesticides	kg ha^{-1}			13,77	137,70	137,70	137,70	137,70	137,70	137,70	137,70	137,70	137,70	137,70	137,70	137,70
Irrigation expenses	m³			0,03	11,5	6,1	13,5	278,1	147,7	324,3	278,1	147,7	324,3	278,1	147,7	324,3
Electricity used	h				74,5	59,2	43,9	6,8	74,5	59,2	43,9	6,8	74,5	59,2	43,9	6,8
Electricity expenses	kWh			0,11	8,0	6,4	4,7	0,7	8,0	6,4	4,7	0,7	8,0	6,4	4,7	0,7
**Input expenses**					1914,42	1907,36	5011,33	7038,07	6914,96	7089,95	9514,62	9380,21	9564,15	3864,71	3732,65	3905,29
**Labour**																
Plowing	ha	10,0		4,02	40,17	40,17	40,17	40,17	40,17	40,17	40,17	40,17	40,17	40,17	40,17	40,17
Rotary tiller	ha	10,0		3,60	36,03	36,03	36,03	36,03	36,03	36,03	36,03	36,03	36,03	36,03	36,03	36,03
Planting	ha	10,0		3,44	34,37	34,37	34,37	34,37	34,37	34,37	34,37	34,37	34,37	34,37	34,37	34,37
Inter-row cultivation	ha	10,0		3,35	33,54	33,54	33,54	33,54	33,54	33,54	33,54	33,54	33,54	33,54	33,54	33,54
Agricultural pest control	ha	10,0		3,12	31,15	31,15	31,15	31,15	31,15	31,15	31,15	31,15	31,15	31,15	31,15	31,15
Irrigation and fertilization labor	ha	10,0		3,27	32,71	32,71	32,71	32,71	32,71	32,71	32,71	32,71	32,71	32,71	32,71	32,71
Facility maintenance and repair	ha	10,0		3,27	32,71	32,71	32,71	32,71	32,71	32,71	32,71	32,71	32,71	32,71	32,71	32,71
Harvest	ha	10,0		7,05	70,51	70,51	70,51	70,51	70,51	70,51	70,51	70,51	70,51	70,51	70,51	70,51
**Labor expenses**					311,19	311,19	311,19	311,19	311,19	311,19	311,19	311,19	311,19	311,19	311,19	311,19
**Input and labor expenses**					2225,60	2218,54	5322,52	7349,25	7226,14	7401,13	9825,81	9691,40	9875,34	4175,90	4043,84	4216,48
Revolving fund interest					241,11	240,34	576,61	796,17	782,83	801,79	1064,46	1049,90	1069,83	452,39	438,08	456,78
**Variable Expenses**	$				2466,71	2458,89	5899,13	8145,42	8008,97	8202,92	10890,27	10741,30	10945,17	4628,29	4481,92	4673,26
Unexpected expenses					3,07	3,06	7,34	10,14	9,97	10,21	13,55	13,37	13,62	5,76	5,58	5,81
Field rent	ha	10,0			0	0	0	0	0	0	0	0	0	0	0	0
Depreciation allowance for irrigatic	ha	10000-18000			20,74	20,74	20,74	20,74	20,74	20,74	20,74	20,74	20,74	20,74	20,74	20,74
**Fixed Expenses**					23,81	23,80	28,08	30,87	30,70	30,95	34,29	34,10	34,36	26,50	26,32	26,55
**Total Production Costs**	$				2490,52	2482,68	5927,20	8176,29	8039,68	8233,87	10924,56	10775,41	10979,53	4654,78	4508,24	4699,81
Green forage yield	kg ha^{-1}				84420	112330	57800	86700	96800	76200	93100	78200	87530	29500	36700	24800
Production cost	$ ha^{-1}				0,03	0,02	0,10	0,09	0,08	0,11	0,12	0,14	0,13	0,16	0,12	0,19
Selling price	$ ha^{-1}	10			0,28	0,28	0,28	0,28	0,28	0,28	0,28	0,28	0,28	0,28	0,28	0,28
Gross production value	$				23284,65	30982,77	15942,35	23913,52	26699,29	21017,42	25678,76	21569,06	24142,45	8136,67	10122,56	6840,32
Gross profit	$				20817,94	28523,88	10043,22	15768,10	18690,32	12814,50	14788,49	10827,76	13197,28	3508,38	5640,64	2167,05
Net profit	$				20794,14	28500,08	10015,14	15737,23	18659,62	12783,55	14754,20	10793,65	13162,92	3481,88	5614,33	2140,50
Relative profit	%				8,35	11,48	1,69	1,92	2,32	1,55	1,35	1,00	1,20	0,75	1,25	0,46

N_0: No nitrogen application, N_1: 140 kg N ha^{-1} nitrogen application; N_2: 210 kg N ha^{-1} nitrogen application; N_3: 280 kg N ha^{-1} nitrogen application, I_1:full irrigation; 100%, I_2:deficit irrigation; 50% of I_1, and I_3:excess irrigation, 120% of I_1. The USD value used in the economic analysis refers to the years 2022 and 2023, when the research was conducted. In 2022, 1 USD was equal to 16.59 Turkish Lira, and in 2023 it was equal to 24.11 Turkish Lira.
